# Efficacy of *AloeVera* Cream in the Treatment of *Paederus* Dermatitis in Mice

**Published:** 2017-05-27

**Authors:** Ramin Khaghani, Iraj Mirzaii-Dizgah, Mostafa Ghasemi

**Affiliations:** 1 Department of Medical Parasitology, School of Medicine, Aja University of Medical Sciences, Tehran, Iran; 2 Department of Physiology, School of Medicine, Aja University of Medical Sciences, Tehran, Iran

**Keywords:** *Paederus*, Dermatitis, *Aloevera*

## Abstract

**Background:**

Dermatitis caused by *Paederus* beetle involves many people around the world, especially Iran. The symptoms include redness, itching and severe irritation. This study evaluated the effectiveness of the *Aloevera* cream on the treatment of dermatitis caused by *Paederus* beetles.

**Methods:**

Forty male 6–8 weeks BALB/C mice were randomly divided into four groups of 10 mice. After removing the mice’s back hair, the backs of mice were marked by *a circle* with a *diameter* of 3 mm. The *Paederus* beetles were collected from Babol in Mazandaran Province, northern Iran and transferred to the animal lab of Aja University of Medical Sciences, Tehran, Iran. The end of abdominal segment *Paederus* was cut with scissors and hemolymph content was pushed by forceps on the circle. Only hemolymph of one *Paederus* applied to the back of each mouse. Groups 1, 2, 3 and 4 were treated with the base (vehicle), dexamethasone 0.1%, *Aloevera* 0.5% and *Aloevera* 2% creams respectively. After 2 days, dermatitis appeared. Then the mentioned creams were applied on the mice once a day. The wound area was measured every day. Dermatitis surface area under curve (AUC) of each mouse was calculated for 17 days after induction of dermatitis. Statistical analysis of ANOVA was used.

**Results:**

Application of *Aloevera* 0.5% and 2% significantly reduced the healing duration and dermatitis area in comparison with the vehicle and dexamethasone cream (P< 0.05). But dexamethasone had no significant effect on the healing of dermatitis as compared to vehicle.

**Conclusion:**

*Aloevera* may clinically effective in the treatment of *Paederus* dermatitis.

## Introduction

The genus *Paederus* is related to family *Staphyllinidae*, order *Coleoptae*, *class Insecta* and consists of over 622 species distributed all around the world (Kerdel-Vegas and Goihman-Yahr 1966, Zargari et al. 2003). Adult rove beetles are between 7–10mm long and 0.5mm wide. These beetles have a black head, a red thorax, upper abdomen, elytral and a lower abdomen (Frank and Kanamitsu 1987, George and Hart 1990). Many species of the rove beetle genus *Paederus* contain the hemolymph toxin named pederin (Frank and Kanamitsu 1987). This substance causes itching and skin lesions in humans. With their hemolymph penetrated into the skin, these results in an affliction called dermatitis linearis. The severe cytotoxic effects of pederin are based on blocking of protein synthesis and inhibition of mitosis in eukaryotic cells (Brega et al. 1968).

This dermatitis is more frequent on the uncovered parts of the body like face, neck and hands. The acute lesions become crusted within a few days and heal completely in about 2 to 3 weeks and may leave a hyperpigmentation (Kerdel-Vegas and Goihman-Yahr 1966, Gelmetti and Grimalt 1993).

Recently, the production of pederin is depended on the activities of an endosymbiont (*Pseudomonas species*) within *Paederus*. The manufacture of pederin is mostly confined to adult females. Larva and males store pederin in very small quantities compared to females (Piel 2002).

*Aloevera* (family: *Liliaceae*) has been used in traditional medicine since ancient times. It is one of the most useful herbs in the world and the medicinal part is the leaves. *Aloevera* leaves contain various essential nutrients for the body, such as amino acids, B family vitamins, etc. It also has pharmacological effects like antioxidant, wound healing, antifungal, antibacterial and immunomodulatory effects (Rodriguez-Bigas et al. 1988, West and Zhu 2003, Hu et al. 2005, Tai-Nin Chow et al. 2005, Olaleye et al. 2005, Rosca-Casian et al. 2007, Habeeb et al. 2007, Maenthaisong et al. 2007).

*Aloevera* also has anti-inflammatory properties (Reynolds and Dweck 1999, Vogler and Ernst 1999). Compounds existing in the inner gel including salicylates, magnesium lactate, bradykinin, thromboxane inhibitors, sterols and a beta linked acetyl mannan (acemannan) have demonstrated anti-inflammatory activity (Vázquez et al. 1996, Talmadge et al. 2004). The mucilaginous polysaccharides in the pulp of *Aloevera* leaf are the major ingredient that induces regeneration. Emodin - as a derivative of anthraquinones produced by pericyclic cells on the surface of *Aloe* leaves- can also promote the healing of rats’ excisional wounds by stimulating regeneration of cells (Eshun et al. 2004, Tang et al. 2007). These researches support the claim that the healing properties of *Aloe* are essentially result of the synergistic mode of action of many bioactive ingredients of *Aloe* leaves (Dagne et al. 2000).

The purpose of the present study was to determine the efficacy of *Aloevera* in the treatment of dermatitis caused by *Paederus* beetles.

## Materials and Methods

### Animals

Forty male BALB/C mice (6–8 weeks old) were obtained from Pasteur Institute of Iran. Animals had free access to food and water and were kept at room temperature and with an artificial light cycle of 12-h light and 12-h dark.

All animal care and procedures and handling were performed under supervision of Animal Care and Use Committee of the Aja University of Medical Sciences.

### Chemicals

Dexamethasone acetate (CAS Number: 464-92-6) was purchased from Chemos GmbH (Regenstauf, Germany). All other chemicals used in this study were of analytical grade and obtained from Merck (Darmstadt, Germany).

### Aloe vera

The pure spray-dried *Aloevera* powder was purchased from Anamis Company (Alborz, Iran). This product consists of the inner gel of the leaves of *Aloevera*.

### *Paederus* Collection and Identification

The *Paederus* beetles were collected from Babol in Mazandaran Province of Iran and transferred to the animal lab of Aja University of Medical Sciences, Tehran, Iran. Identification was made by specialists and by using the key and illustrations of Coiffait (1982). *Paederus* beetles were identified as *Paederusfuscipes* that together with *Pa. kalalovae* are the predominant species along the southern shores of the Caspian Sea (Nikbakhtzadeh and Tirgari 2008).

### Dermatitis induction and treatments

Forty male 6–8 weeks BALB/C mice were randomly divided into four groups (10 mice in each group). Each mouse was shaved on the back (2cm× 2cm) and the skin was gently wiped with distilled water. The backs of mice were marked by a circle with a diameter of 3 millimeters. The end of abdominal segment of adult female *Paederus* beetles was cut with scissors and hemolymph content was push by forceps on the circle. Only hemolymph of one *Paederus* applied to the back of each mouse.

**Fig. 1.**
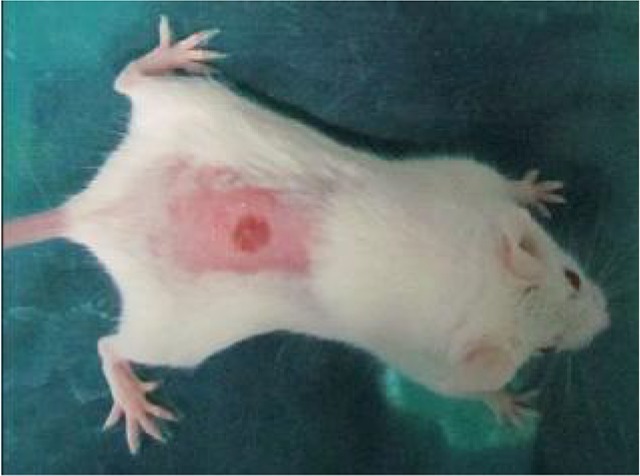
Dermatitis induced by Paederus hemolymph on the back of mouse.

After 48 h the dermatitis appeared clearly (Fig. 1) and its surface area was measured and recorded as per square millimeter (mm^2^). Groups 1, 2, 3 and 4 were treated with the base cream (as vehicle group), dexamethasone 0.1 % cream, *Aloevera* 0.5% cream and *Aloevera* 2% cream respectively. Drug doses had been shown in a previous study to be adequate to elicit a response (Babaee et al. 2012)

### Preparation of the creams

Dexamethasone and *Aloevera* powder were formulated in an aqueous cream containing polysorbate 80 (5w/v), Isopropyl myristate, stearyl alcohol (20v/v), liquid white paraffin (15v/v), purified water (100ml), methylparaben (0.18w/w) and propylparaben (0.02w/w). Preparation process carried out under conditions in which dexamethasone and *Aloevera* powder were not heated. The creams contained *Aloevera* powder, dexamethasone cream and base cream were prepared according to similar protocol, under the same conditions and with the same chemicals.

### Dermatitis surface area measurement

To determine the surface areas of dermatitises, the mice were observed every day. A photograph was taken of each mouse’s dermatitis with a single lens, 14.1MP Digital Camera (SX30 IS, Canon, Japan). For calibrating the magnification of photos, camera’s lens was held at a distance of 20cm from the dermatitis and a caliper was placed at the level of mice’s back. The wound area was measured per mm^2^ every day by analysis of images using Image J (Image Processing and Analysis in Java, US National Institutes of Health) and Adobe Photoshop CS5 software. Dermatitis surface area under curve (AUC) of each mouse was calculated for 17d after induction of dermatitis (dermatitis surface in mm^2^ × time between observations in day).

### Statistical analysis

Values are expressed as Mean± SEM. One-way analysis of variance, ANOVA (post hoc Tukey test) was used to determine significant differences among groups and P< 0.05 was considered a significant difference. Statistical analysis performed using SPSS software (version 15, SPSS Inc., Chicago, IL, USA).

## Results

In the second day after dermatitis induction, there was no significant difference in the dermatitis surface area values among groups. The mean (± SEM) dermatitis surface areas were 19.61±0.43mm^2^, 19.89±0.43mm^2^, 19.34± 0.46mm^2^ and 19.5±0.51mm^2^ for group 1 to 4, respectively.

**Fig. 2.**
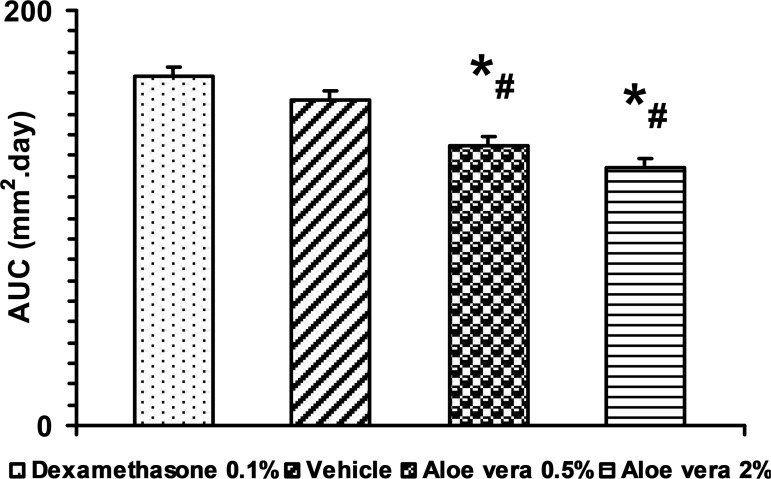
Effect of treatment with base cream (vehicle), dexamethasone 1% or Aloe vera cream 0.5 and 2% on dermatitis surface changes for 17 day observations (as indicated by area under curve (AUC)) following exposure to *Paederus*’ hemolymph in BALB/C mice. Data are expressed as mean ± SEM. * and # different from vehicle and dexamethasone 0.1% treated, respectively, P< 0.05.

A one-way ANOVA indicated that the means AUC of the dermatitis surface was significantly different among groups (P< 0.05, Fig. 2). Post-hoc analysis showed that *Aloevera* treatment dose dependently decreased the AUC of the dermatitis surface as compared to vehicle and dexamethasone treatments (P< 0.05). There was no significant difference between vehicle and dexamethasone treatments and also between two doses of *Aloevera* creams (P> 0.05).

**Fig. 3.**
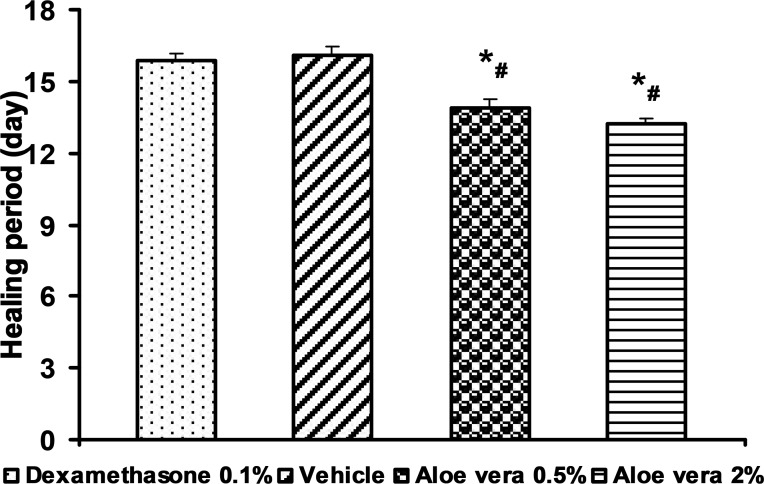
Effect of treatment with base cream (vehicle), dexamethasone 1% or *Aloe vera* cream 0.5 and 2% on the days passed for completing healing induced by *Paederus*’ hemolymph in BALB/C mice. Data are expressed as mean ± SEM. * and # different from vehicle treated (vehicle) and dexamethasone 0.1 % treated, respectively, P< 0.05.

*Aloevera* creams (0.5% and 2%) significantly reduced the healing period of dermatitis in comparison with dexamethasone and base creams (groups 1 and 2) (P< 0.05, Fig. 3). But there was no significant difference between two *Aloevera* creams. Dexamethasone cream and vehicle had no significant difference in reduction of healing duration of dermatitis (Fig. 3). Healing time ranged from 14 to 17 days in vehicle group, 15 to 17 days in dexamethasone 1% treated group, 13 to 16 days in *Aloe vera* 0.5% treated group and 12 to 14 days in *Aloe vera* 2% treated group.

## Discussion

Dermatitis caused by *Paederus* beetle involves many people around the world, especially in Iran. The symptoms include redness, itching and severe irritation. In this study the efficacy of the *Aloevera* cream on the treatment of dermatitis caused by *Paederus* beetles was investigated. *Aloevera* creams (0.5 and 2 percent) decreased the healing period of dermatitis and AUC of the dermatitis surface. Thus it seems that *Aloevera* creams (0.5 and 2 percent) have capability to promote dermatitis healing more effectively than dexamethasone and base creams.

Up to now, numerous studies have been performed on the effects of *Aloe vera.* Although there are many studies that have shown the efficacy of different *Aloe vera* preparation on various dermatologic disorders such as fungal and bacterial infections, incision and burn wounds, skin inflammations and many others (Rodriguez-Bigas et al. 1988, Vázquez et al. 1996, Reynolds and Dweck 1999, Vogler and Ernst 1999, Yagi et al. 2002, Rosca-Casian et al. 2007, Maenthaisong et al. 2007), but its effect on dermatitis linearis caused by pederin existing in Paederus’ hemolymph was unknown.

*“Aloe vera* mouthwash was effective in healing of the wound and reducing the inflammation of the mucous membrane of the mouth” (Mansour et al. 2013). The topical application of *Aloe vera* improves the total quality of life score in patients with oral lichen planus (Salazar-Sánchez et al. 2010). Furthermore, its gel has been effective in healing of the first and second degree burning wounds without any side effects (Maenthaisong et al. 2007) and also in cesarean wound healing (Tarameshloo et al. 2012, Molazem et al. 2014). *Aloe vera* 2% oral gel is effective in decreasing the recurrent aphthous stomatitis patients’ pain score and wound size and decreases the aphthous wound healing period too (Babaee et al. 2012). *Aloe vera* reduces inflammatory cytokines (such as IL-18), INF-α neutrophil chemoattractant, and malondialdehyde as indicator of oxidative stress (Werawatganon et al. 2014).

Many of the biological activities of *Aloe vera* such as immunostimulation, anti-inflammatory effects, wound healing, promotion of radiation damage repair, anti-bacterial, anti-viral, anti-fungal, anti-diabetic and anti-neoplastic activities, and stimulation of hematopoiesis and anti-oxidant effects have been attributed to the polysaccharides contained in the gel of the leaves. These biological activities should be assigned to a synergistic action of the compounds contained therein rather than a single chemical substance (Hamman 2008).

Results of our study showed that there was no significant difference between dexamethasone cream and vehicle in reducing the dermatitis healing duration. This is in agreement with Qadir et al. report (2006) that combination of topical steroids and oral antibiotics have better effect than topical steroids alone on treatment of *Paederus* dermatitis in Sierra Leone patients. Cortisone or antihistamine preparations have no beneficial effect (Deneys and Zumpt 1963). As dexamethasone is 25 times more potent than cortisol in its glucocorticoid effect, it seems that due to anti-inflammatory and immunosuppressant effects of glucocorticoids, without antibiotic medication, these medications can be even harmful by favoring secondary infections.

## Conclusion

*Aloevera* seems to be clinically effective in the treatment of *Paederus* dermatitis. It may be considered as an alternative or supplementary medicine for patients with this disease. Further investigation on human and prolonged follow-up period is proposed in order to confirm the efficacy of *Aloevera* in the treatment of *Paederus* dermatitis in human.
